# Supplementary figures and images for: The Caenorhabditis elegans homolog of the Evi1 proto-oncogene, egl-43, coordinates G1 cell cycle arrest with pro-invasive gene expression during anchor cell invasion
Source: PLoS Genet. 2020 Mar 23;16(3):e1008470. doi: 10.1371/journal.pgen.1008470 (PMC7117773; doi:10.1371/journal.pgen.1008470)

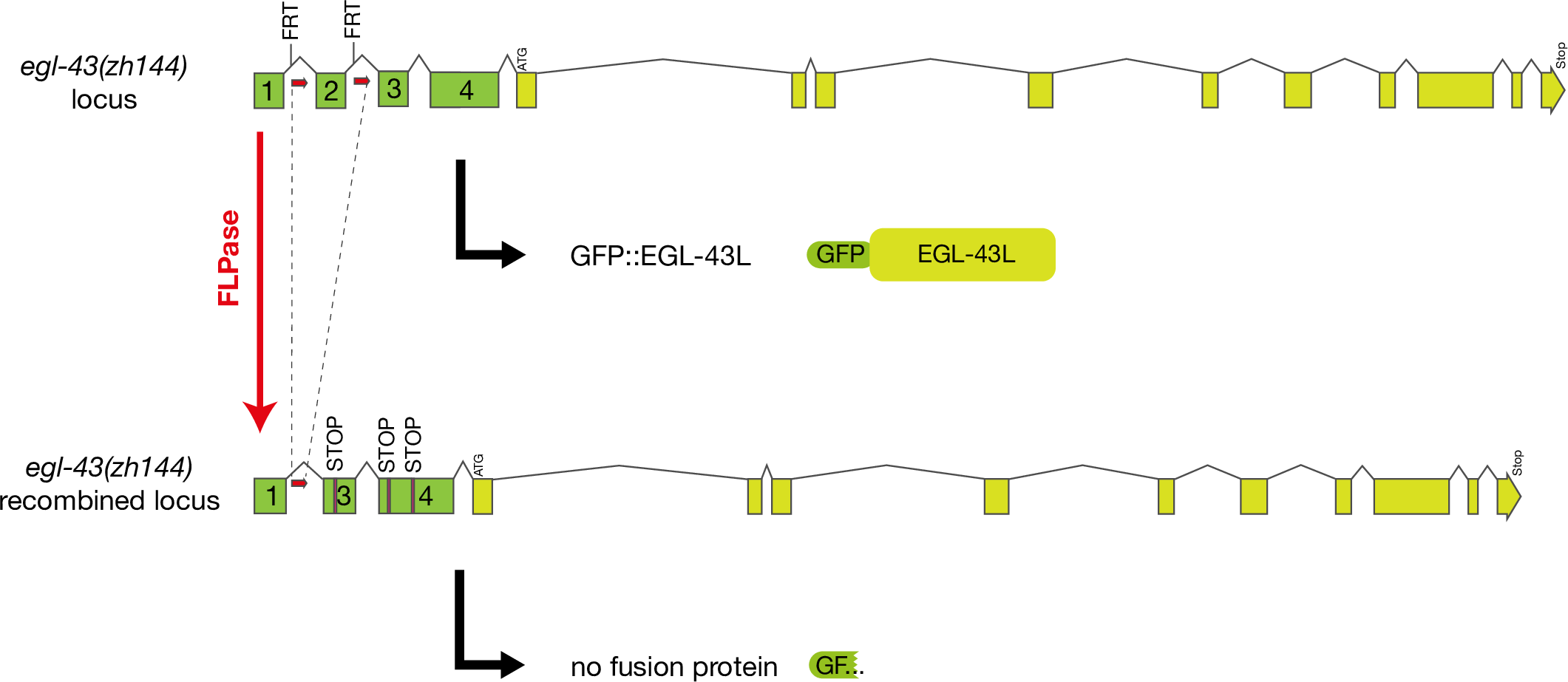

Supplement: S1 Fig — (TIF) [file pgen.1008470.s001.tif]

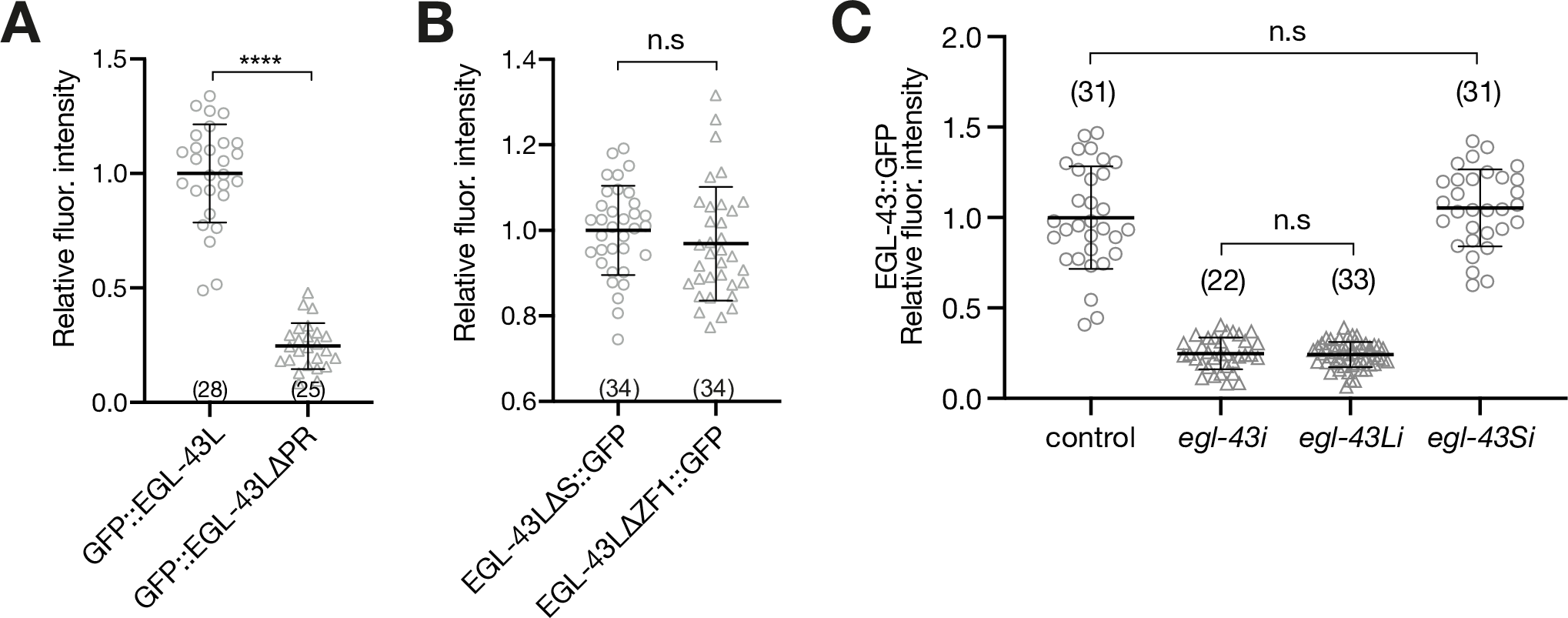

Supplement: S2 Fig — (A) Quantification of GFP::EGL-43L and GFP::EGL-43LΔPR and (B) EGL-43LΔS::GFP and EGL-43LΔZF1::GFP expression levels in the AC of mid-L3 larvae. N- and C-terminal GFP fusions were quantified separately because the site of insertion potentially affects GFP signal intensity. (C) Quantification of the AC expression levels of EGL-43::GFP, a reporter for both the EGL-43L and EGL-43S isoforms, upon control, egl-43, egl-43L, and egl-43S RNAi. The numbers of animals analyzed for each condition are shown in bracket. The error bars indicate standard deviations and the horizontal bars the mean values. Statistical significance was determined by Student’s t-tests and is indicated with n.s. for p>0.05 and **** for p<0.0001. (TIF) [file pgen.1008470.s002.tif]

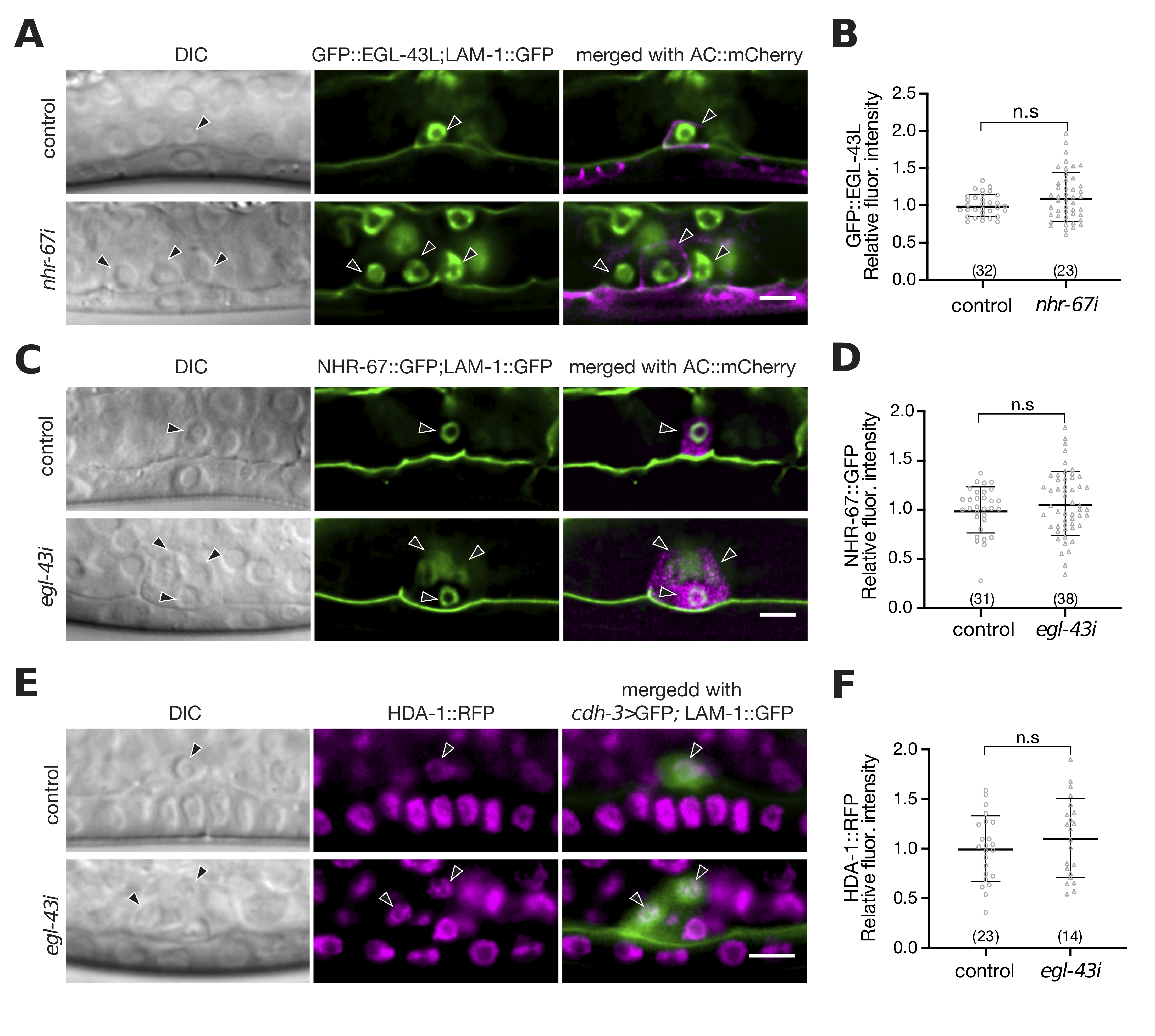

Supplement: S3 Fig — (A) NHR-67::GFP expression in the ACs of early-L3 larvae (Pn.p stage) after egl-43 RNAi. (B) Quantification of NHR-67::GFP expression levels after egl-43 RNAi. (C) GFP::EGL-43L expression in the ACs of early-L3 larvae after nhr-67 RNAi. (D) Quantification of GFP::EGL-43 expression levels after nhr-67 RNAi. (E) HDA-1::RFP expression in mid-L3 larvae after egl-43 RNAi. (F) Quantification of the HDA-1::RFP expression shown in (E). For all reporters, left panels show Nomarski (DIC) images, middle panels the respective reporter together with the LAM-1::GFP BM marker, and right panels merged images with the ACs labelled by cdh-3>mCherry::PH (A), cdh-3>mCherry::moeABD (C) or cdh-3>gfp (E). The error bars indicate standard deviations and the horizontal bars the mean values. Statistical significance was determined with a Student’s t-test and is indicated with ** for p<0.01 and n.s. for p>0.05. The numbers in brackets refer to the numbers of animals analyzed. The scale bars are 5 μm. (TIF) [file pgen.1008470.s003.tif]

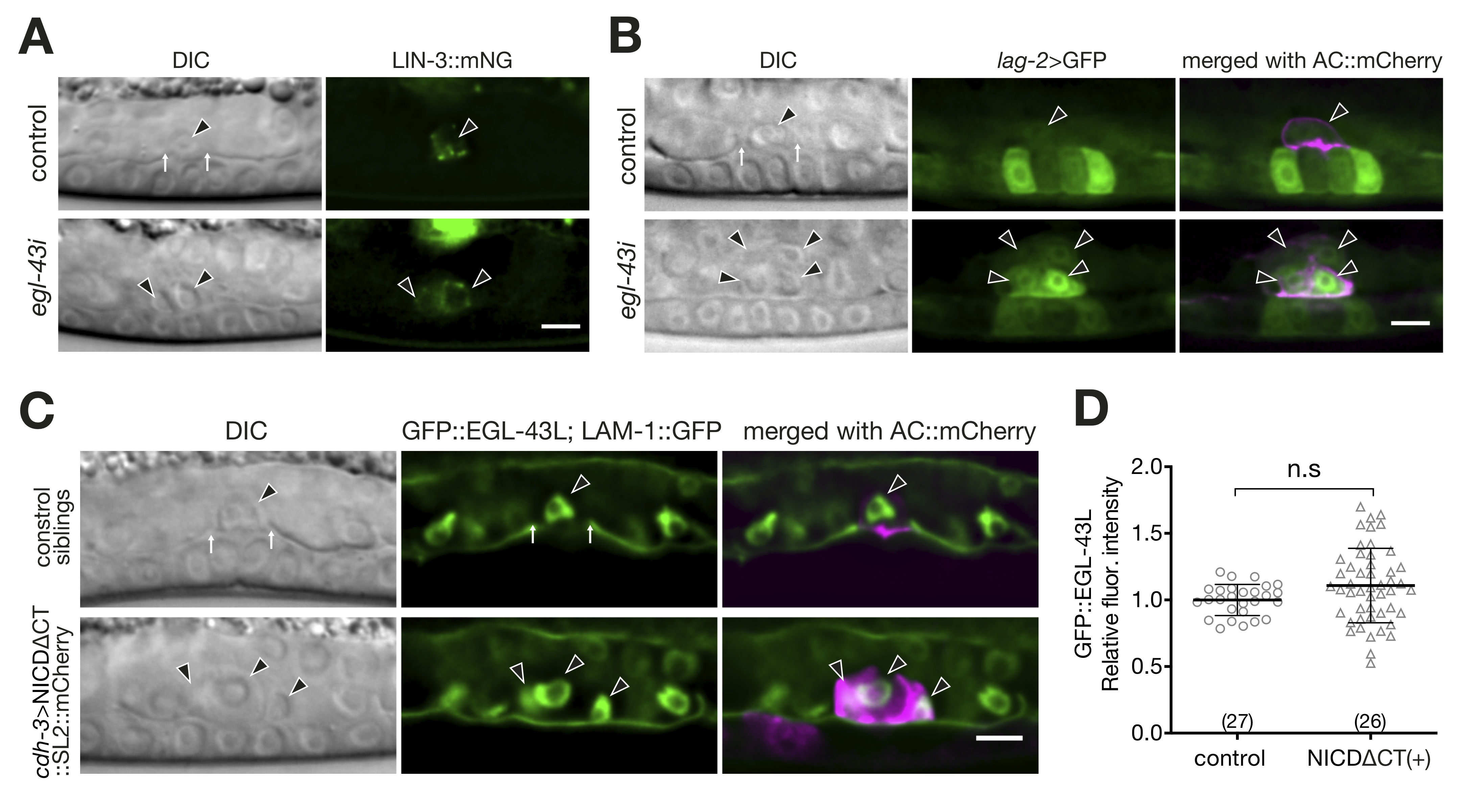

Supplement: S4 Fig — (A) lin-3 reporter expression in the control and egl-43 RNAi ACs. Left panels show Nomarski (DIC) images and right panels the fluorescence image with LIN-3::mNG. (B) lag-2 expression in the ACs of control and egl-43 RNAi. Left panels shows Nomarski (DIC) images, middle panel the fluorescence image with lag-2>GFP reporter, and right panels the reporter merged with AC marker cdh-3>mCherry::PH. (C) GFP::EGL-43L expression in the ACs of control and NICDΔCT expressing ACs. Left panels show Nomarski (DIC) images, middle panels the GFP::EGL-43 signal with the LAM-1::GFP BM marker, and right panels merged with the ACs labelled with cdh-3>PH::mCherry (control, row 1) and cdh-3>NICDΔCT::SL2::mCherry (row 2) respectively. (D) Quantification of the GFP::EGL-43L expression shown in (C). The error bars indicate standard deviations and the horizontal bars the mean values. Statistical significance was determined with a Student’s t-test and is indicated with n.s. for p>0.05. The numbers in brackets refer to the numbers of animals analyzed. The scale bars are 5 μm. (TIF) [file pgen.1008470.s004.tif]

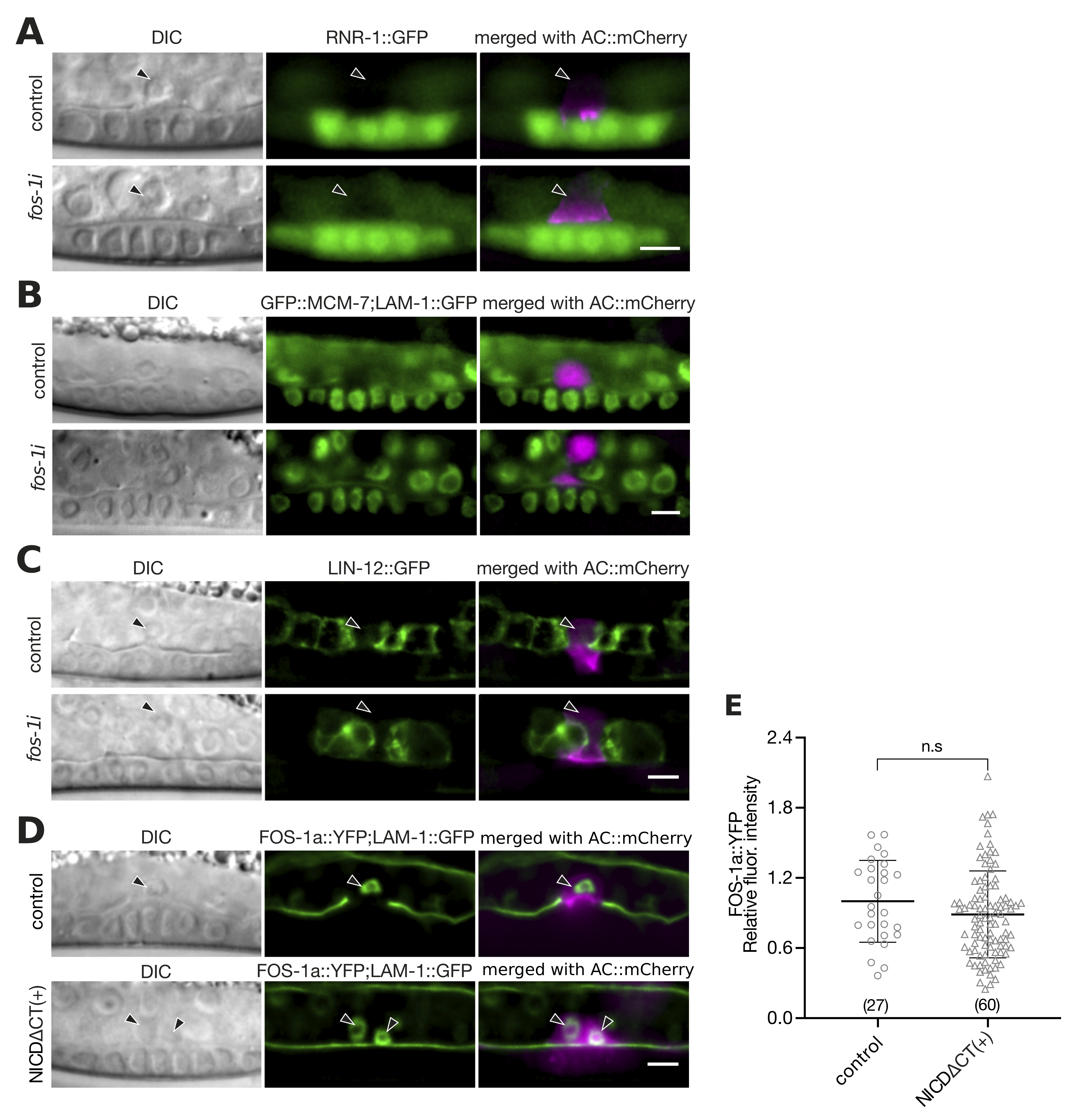

Supplement: S5 Fig — (A) Expression of the S-phase marker RNR-1::GFP after control and fos-1 RNAi. None of 19 control or 23 fos-1i animals showed RNR-1::GFP expression in the AC. (B) Expression of GFP::MCM-7 after control and fos-RNAi. None of 25 control or 25 fos-1i animals showed GFP::MCM-7 expression. (C) LIN-12::GFP expression is not up-regulated after control (0/20) or fos-1 (0/24) RNAi treatment. (D) Expression of FOS-1a::YFP in control and NICDΔCT-expressing ACs of mid-L3 larvae. (E) Quantification of the FOS-1a::YFP expression shown in (D). For each reporter, the left panels show Nomarski (DIC) images, the middle panels the GFP or YFP signals of the indicated reporters in green (in (B) and (D) together with the LAM-1::GFP BM marker) and the right panels the GFP reporter signals merged with the ACs labelled with the cdh-3>mCherry::moeABD (A, C), lin-3ACEL>mCherry (B) or cdh-3>nicdΔct::sl2::mCherry (D) reporters in magenta. The black arrowheads point at the AC nuclei and the white arrows at the locations of the BM breaches. The error bars indicate standard deviations and the horizontal bars the mean values. Statistical significance was determined with a Student’s t-test and is indicated with n.s for p>0.05. The numbers in brackets refer to the numbers of animals analyzed. The scale bars are 5 μm. (TIF) [file pgen.1008470.s005.tif]
