# Supplementary material for: The Caenorhabditis elegans homolog of the Evi1 proto-oncogene, egl-43, coordinates G1 cell cycle arrest with pro-invasive gene expression during anchor cell invasion
Source: PLoS Genet. 2020 Mar 23;16(3):e1008470. doi: 10.1371/journal.pgen.1008470 (PMC7117773; doi:10.1371/journal.pgen.1008470)
Supplement: S1 Table — (DOCX) [file pgen.1008470.s006.docx]

| **Strain** | **Genotype** |
| --- | --- |
| AH3438 | *maIs103[rnr::gfp]; rrf-3(pk1426)II; qyls10[lam-1::GFP]IV; qyIs50[cdh-3>mCherry::moeABD]V* |
| AH4566 | *zh112[lin-3::mNeongreen::LoxP::3xFlag]IV* |
| AH4743 | *qyIs23[cdh-3>mCherry::PH]II; zh118[GFP(frt)::mcm-7]V; qyIs10[lam-1::GFP]IV* |
| AH4758 | *zhIs131[cyd-1>cyd-1(exon1,2)::gfp]II* |
| AH5218 | *zhIs127[lin-3^ACEL^>mCherryI; rrf-3(pk1426) II;qyIs10[lam-1::gfp] IV; zh118[gfp-frt::mcm-7]V* |
| AH5589 | *wgIs373[nhr-67::TY1::egfp::3xFLAG]; zhIs127[lin-3^ACEL^>mCherry]I; qyIs10[lam-1::gfp]IV* |
| AH5620 | *zhIs127[lin-3^ACEL^>mCherry]I; Zh136[egl-43::ZF1::gfp] II; rrf-3(pk1426) II; qyIs10[lam-1::gfp] IV* |
| AH5654 | *zh146[ΔFRE>gfp::egl-43L]II; qyIs23[cdh-3>mCherry::PH]II; qyIs10[lam-1::gfp]IV* |
| AH5655 | *zh144 [gfp::egl-43L]II; qyIs23[cdh-3>mCherry::PH]II; qyIs10[lam-1::gfp]IV* |
| AH5705 | *qyIs23[cdh-3>mCherry::PH]II; qIs56[lag-2>gfp]V* |
| AH5706 | *wgIs72[lin-12::TY1::egfp]; qyIs23[cdh-3>mCherry::PH]II* |
| AH5717 | *zh144[gfp::egl-43L]II;qyIs23[cdh-3>mCherry::PH]II; qyIs10[lam-1::gfp]IV; fos-1(ar105)V/nT1* |
| AH5718 | *zh146[ΔFRE>gfp::egl-43L]II; qyIs23[cdh-3>mCherry::PH]II; qyIs10[lam-1::gfp]IV; fos-1(ar105) V/nT1* |
| AH5748 | *zh149[egl-43LΔS::gfp]II; qyIs10[lam-1::gfp] IV* |
| AH5754 | *syIs118[fos-1a::yfp-TX]I; qyIs23[cdh-3>mCherry::PH]II; qyIs10[lam-1::gfp] IV* |
| AH5755 | *qyIs23[cdh-3>mCherry::PH] II; qyIs10[lam-1::gfp] IV* |
| AH5756 | *zhIs130[lin-3^ACEL^>CDK sensor::egfp] I; qyIs50[cdh-3>mCherry::moeABD] V; rrf-3(pk1426)II; qyIs10[lam-1::gfp]IV* |
| AH5757 | *zh148[egl-43>gfp::SEC]II/mln1; qyIs50[cdh-3>mCherry::moeABD] V* |
| AH5761 | *zh148[gfp::egl-43LΔPR]II; qyIs23[cdh-3>mCherry::PH]II; qyIs10[lam-1::gfp]IV* |
| AH5777 | *zhEx647[cdh-3>nicdΔCT::SL2::mCherry]; qyIs23[cdh-3>mCherry::PH]II; qyIs10[lam-1::GFP]IV* |
| AH5778 |  |
| AH5779 |  |
| AH5780 | *zhEx655[lin-3^ACEL^>flp-D5]; zh144[gfp::egl-43L]II; qyIs23[cdh-3>mCherry::PH]II; qyIs10[lam-1::gfp]IV* |
| AH5781 |  |
| AH5783 | *zhEx656[cdh-3>mCherry]; qyIs23[cdh-3>mCherry::PH]II; qyIs10[lam-1::gfp]IV* |
| AH5783 |  |
| AH5784 |  |
| AH5787 | *zhEx657[cdh-3>cki-1::SL2::mNG]; qyIs23[cdh-3>mCherry::PH]II; qyIs10[lam-1::gfp]IV* |
| AH5789 | *zh150[egl-43LΔZF1::gfp]II; qyIs10[lam-1::gfp]IV* |
| AH5800 | *zhEx647[cdh-3>nicdΔCT::SL2::mCherry]; zh144 [gfp::egl-43L]II; qyIs23[cdh-3>mCherry::PH]II; qyIs10[lam-1::gfp]IV* |
| AH5810 | *zhEx647[cdh-3>nicdΔCT::SL2::mCherry]; qyIs23[cdh-3>mCherry::PH]II; zh118[gfp(frt)::mcm-7]V; qyIs10[lam-1::GFP]IV* |
| AH5860 | *zhEx647[cdh-3>nicdΔCT::SL2::mCherry]; syIs118[ fos-1a::yfp-TX]; qyIs23[cdh-3>mCherry::PH]II; qyIs10[lam-1::GFP]IV* |
