# Supplementary material for: The Caenorhabditis elegans homolog of the Evi1 proto-oncogene, egl-43, coordinates G1 cell cycle arrest with pro-invasive gene expression during anchor cell invasion
Source: PLoS Genet. 2020 Mar 23;16(3):e1008470. doi: 10.1371/journal.pgen.1008470 (PMC7117773; doi:10.1371/journal.pgen.1008470)
Supplement: S2 Table — (DOCX) [file pgen.1008470.s007.docx]

| **Construct** | **Plasmid** | **PCR amplification** | | | | | **Vector backbone** |
| --- | --- | --- | --- | --- | --- | --- | --- |
|  |  |  | Fragment | Fw Primer | Rv Primer | Template |  |
| *lin-3^ACEL^>flp-D5* | pEH4 | PCR1 | *lin-3^ACEL^* | oTD70 | oTD71 | pTD14 | pWD79-2RV (PvuII+MluI) |
|  |  | PCR2 | *2XNLS-flp-D5* | oTD72 | oTD73 | pMLS262 |  |
| *egl-43L RNAi* | pEV48 | PCR | *egl-43L* | oEL316 | oEL317 | N2 genomic DNA | L4440  (PstI/XhoI) |
| *egl-43S RNAi* | pEV49 | PCR | *egl-43S* | oEL318 | oEL319 | N2 genomic DNA | L4440  (PstI/XhoI) |
| *gfp(frt)::mcm-7* | pTD16 | PCR1 | 5' homology arm | oTD140 | oTD141 | N2 genomic DNA | pMW75 (ClaI+SpeI) |
|  |  | PCR2 | 3' homology arm | oTD142 | oTD143 | N2 genomic DNA |  |
| *egl-43::gfp* | pTD24 | PCR1 | 5' homology arm | oTD257 | oTD258 | N2 genomic DNA | pMW75 (ClaI+SpeI) |
|  |  | PCR2 | 3' homology arm | oTD259 | oTD260 |  |  |
| *lin-3^ACEL^-∆pes10> CDK sensor::egfp* | pTD30 | PCR1 | *lin-3^ACEL^* promoter | oTD277 | oTD136 | pTD14 | pCFJ151 (AvrII/SpeI) |
|  |  | PCR2 | CDK sensor::egfp | oTD278 | oTD279 | SV1667 genomic DNA |  |
| *gfp::egl-43L* | pTD32 | PCR1 | 5' homology arm | oTD311 | oTD312 | N2 genomic DNA | pMW75 (ClaI+SpeI) |
|  |  | PCR2 | 3' homology arm | oTD313 | oTD314 |  |  |
| *ΔFRE>gfp::egl-43L* | pTD34 | PCR1 | fragment 1 | oTD307 | oTD321 | N2 genomic DNA | pMW75 (ClaI+SpeI) |
|  |  | PCR2 | fragment 2 | oTD322 | oTD310 |  |  |
|  |  | PCR3 | 5' homology arm | oTD311 | oTD312 | fragment 1 & 2 |  |
|  |  | PCR4 | 3' homology arm | oTD313 | oTD314 | N2 genomic DNA |  |
| *gfp::egl-43LΔPR* | pTD43 | PCR1 | 5' homology arm | oTD311 | oTD312 | N2 genomic DNA | pMW75 (ClaI+SpeI) |
|  |  | PCR2 | 3' homology arm | oTD326 | oTD327 |  |  |
| *egl-43LΔS::gfp* | pTD46 | PCR1 | 5' homology arm_1 | oTD357 | oTD358 | N2 genomic DNA | pMW75 (ClaI+SpeI) |
|  |  | PCR2 | 5' homology arm_2 | oTD359 | oTD360 | pSS16 |  |
|  |  | PCR3 | 3' homology arm | oTD361 | oTD214 | N2 genomic DNA |  |
| *egl-43LΔZF1::gfp* | pTD51 | PCR1 | 5' homology arm_1 | oTD370 | oTD371 | N2 genomic DNA | pMW75 (ClaI+SpeI) |
|  |  | PCR2 | 5' homology arm_2 | oTD372 | oTD360 | pTD46 |  |
|  |  | PCR3 | 3' homology arm | oTD373 | oTD214 | N2 genomic DNA |  |
| *cdh-3>cki-1*  *::SL2::mNG* | pTD55 | PCR1 | *cdh-3* promoter | oTD402 | oTD286 | pEV27 | pCFJ151 (AvrII/SpeI) |
|  |  | PCR2 | *cki-1* | oTD287 | oTD300 | N2 genomic DNA |  |
|  |  | PCR3 | *SL2::mNG* | oTD301 | oTD302 | pBN396 |  |
| *cdh-3>nicdΔCT*  *::SL2::mCherry* | pTD56 | PCR1 | *cdh-3* promoter | oTD203 | oTD394 | pEV27 | pCFJ151 (AvrII/SpeI) |
|  |  | PCR2 | *nicdΔCT* | oTD395 | oTD396 | pSH16 |  |
|  |  | PCR3 | *SL2::mCherry* | oTD397 | oTD299 | pTD30 |  |
| *cdh-3>mCherry* | pTD57 | PCR1 | *cdh-3* promoter | oTD404 | oTD203 | pTD56 | pCFJ151 (AvrII/SpeI) |
|  |  | PCR2 | *mCherry* | oTD403 | oTD299 | pTD56 |  |
