# Supplementary material for: The Caenorhabditis elegans homolog of the Evi1 proto-oncogene, egl-43, coordinates G1 cell cycle arrest with pro-invasive gene expression during anchor cell invasion
Source: PLoS Genet. 2020 Mar 23;16(3):e1008470. doi: 10.1371/journal.pgen.1008470 (PMC7117773; doi:10.1371/journal.pgen.1008470)
Supplement: S3 Table — (DOCX) [file pgen.1008470.s008.docx]

| **Primer** | **Sequence (5'-3')** |
| --- | --- |
| OEL316 | atcccccgggctgcaGCCGGACAGTACTCAAAG |
| OEL317 | gtaccgggcccccccCACTGAATGAACTCTCCG |
| OEL318 | atcccccgggctgcaGGTGTGTCACAGGGCTCATTC |
| OEL319 | gtaccgggcccccccCTGGAATATTTATTCACTAATGAATAAGACG |
| oTD70 | CGCGCGTTGGCCGATTCATTAATGCAGCTGCACCTGTGTATTTTATGCTGG |
| oTD71 | TTGGCCAATCCCCGGAACACCTGTGTGTACAGTTTTC |
| oTD72 | CTGTACACACAGGTGTTCCGGGGATTGGCCAAAGG |
| oTD73 | AATATACCAAATTGTGGCATTTTTACGCGTAATCAATGCCTGAAAGTTAAAAATTAC |
| oTD136 | GGTACCAGAGCTCACCTAGGCACCTGTGTATTTTATGCTGG |
| oTD140 | GCATCAGTCAGTAGCATCGCACAATTCTACTCTTGAAAC |
| oTD141 | GTTCTTCTCCTTTACTCATGGTGAGTGAGATTATAAGATTATAGAGCCGAGCTG |
| oTD142 | GGATGACGATGACAAGAGAATGAAAACCACCACTTACAACACAGACTTGGCTGCAGAG |
| oTD143 | CAGCTATGACCATGACTAGCATCGTCCGTTGTGAAGTAG |
| oTD203 | gtctagaactagGCCCTAGAGCATGATGTCCTTACC |
| oTD214 | CAGCTATGACCATGACTAGGAGATGGAGAGGTTGGGAGGAG |
| oTD257 | GTAAAACGACGGCCAGTCGCCGGCAGCTCGAGCAGTGGAAGTGG |
| oTD258 | CCTGAGGCTCCCGATGCTCCCACTTTTGGCACTGGAACCGACGT |
| oTD259 | CAAGGATGACGATGACAAGAGATGAGCGCTGCTTCGGTCAG |
| oTD260 | GAAACAGCTATGACCATGTTATGTTTCCGGCCTCGGAACGAC |
| oTD277 | CGTTGGTCATttttGGCGCGCCAATCAATGCCTGAAAGTTAA |
| oTD278 | TTTTAACTTTCAGGCATTGATTGGCGCGCCaaaaATGACC |
| oTD279 | acttaTAATACGACTCACTAGTGTATCTAGAACCGGTGACGTCAC |
| oTD286 | CAACGACGAGCAGAAGACATAGCTCGGTACCCTCCAAG |
| oTD287 | TGCTTGGAGGGTACCGAGCTATGTCTTCTGCTCGTCGTTG |
| oTD299 | cactgatcttacttgcacttaCCGTACGTCTCGAGTGTAAAAC |
| oTD300 | GAAAGTAGGATGAGACAGCTTAGTATGGAGAGCATGAAGATC |
| oTD301 | CTTCATGCTCTCCATACTAAGCTGTCTCATCCTACTTTCAC |
| oTD302 | cactgatcttacttgcacttaTAGAACTAGGCCCGGGG |
| oTD307 | CTCTACCGATTGGCTGATGG |
| oTD310 | GTTGGCGAAGGTACTGTAG |
| oTD311 | GCATCAGTCAGTAGCATCGCTCGCATCCACAAATGGAAG |
| oTD312 | GTTCTTCTCCTTTACTCATCCTGAAAACTTAGGAAACTGTAC |
| oTD313 | GGATGACGATGACAAGAGAATGAGCATCGACACAGACTTTCTCACGAGTGTTGAAGTAAA |
| oTD314 | CAGCTATGACCATGACTAGGGAAAGGTGGAAAGACAGGTG |
| oTD321 | CAGTTTTGGGGCGAAAGATACATCTTGTGAACGAGAAC |
| oTD322 | TCTCGTTCACAAGATGTATCTTTCGCCCCAAAACTG |
| oTD326 | GGATGACGATGACAAGAGAATGGATGCTAATATTTGTATG |
| oTD327 | CAGCTATGACCATGACTAGGGAGATGCTGAAGGATGTGCT |
| oTD357 | GCATCAGTCAGTAGCATCGGCTCCTTAAAGCCGTTTCGG |
| oTD358 | TGGCTGGATCTTTAGGCATCTGGAATATTTATTCACTAATGAAT |
| oTD359 | GTGAATAAATATTCCAGATGCCTAAAGATCCAGCCAAAC |
| oTD360 | GTTCTTCTCCTTTACTCATCTTCACGAACGCCGCCGC |
| oTD361 | GGATGACGATGACAAGAGAGACGGTTGGACGTGCCCAACGTGTCAAAGTCAGCTGCCAT |
| oTD370 | GCATCAGTCAGTAGCATCGGACCTTGAACTTGCCCGATTAG |
| oTD371 | TGGCTGGATCTTTAGGCATCTTGTGTGATCCGTCGGGAG |
| oTD372 | CCGACGGATCACACAAGATGCCTAAAGATCCAGCCAAAC |
| oTD373 | GGATGACGATGACAAGAGAGAGATGACTGCACTCTACAAACC |
| oTD394 | CGACGTTTCCTTGTCATAGCTCGGTACCCTCCAAG |
| oTD395 | TGGAGGGTACCGAGCTATGACAAGGAAACGTCGAATG |
| oTD396 | AAAGTAGGATGAGACAGCTCAGGGTCGAGTTACTTTTC |
| oTD397 | AAGTAACTCGACCCTGAGCTGTCTCATCCTACTTTCAC |
| oTD402 | GAGGGTACCAGAGCTCACTAGAGCATGATGTCCTTACC |
| oTD403 | CTTGGAGGGTACCGAGCTATGGTCTCAAAGGGTGAAGAAG |
| oTD404 | CTTCACCCTTTGAGACCATAGCTCGGTACCCTCCAAG |
