# Supplementary material for: The Caenorhabditis elegans homolog of the Evi1 proto-oncogene, egl-43, coordinates G1 cell cycle arrest with pro-invasive gene expression during anchor cell invasion
Source: PLoS Genet. 2020 Mar 23;16(3):e1008470. doi: 10.1371/journal.pgen.1008470 (PMC7117773; doi:10.1371/journal.pgen.1008470)
Supplement: S4 Table — (DOCX) [file pgen.1008470.s009.docx]

| **Allele** | **Guide RNA sequence** | **Guide RNA name** |
| --- | --- | --- |
| *zh136[egl-43::gfp]* | TCTTCCAGGAACGTCCGTTC | egl-43 sgRNA C1 |
|  | TCACACTTTTGGCACCGGAA | egl-43 sgRNA C3 |
|  | AGCGCTCACACTTTTGGCAC | egl-43 sgRNA C5 |
| *zh144[gfp::egl-43L]* | AGACTTCCTCACGAGTGTTG | egl-43 sgRNA N2 |
|  | GATGCTCATCCTGAAAACTT | egl-43 sgRNA N3 |
| *zh146[ΔFRE>gfp::egl-43L]* | AGACTTCCTCACGAGTGTTG | egl-43 sgRNA N2 |
|  | GATGCTCATCCTGAAAACTT | egl-43 sgRNA N3 |
| *zh148[gfp::egl-43LΔPR]* | AGACTTCCTCACGAGTGTTG | egl-43 sgRNA N2 |
|  | GATGCTCATCCTGAAAACTT | egl-43 sgRNA N3 |
|  | CTGATTAAGGAAGCTGATGA | egl-43 sgRNA PR#2 |
|  | CTCCTGCCTCATACAAATAT | egl-43 sgRNA PR#5 |
| *zh149[egl-43LΔS::gfp]* | AGTGAATAAATATTCCAGGA | egl-43 sgRNA Z2 |
| *zh150[egl-43LΔZF1::gfp]* | AGTGAATAAATATTCCAGGA | egl-43 sgRNA Z2 |
|  | AGTCGGAAAAGTTATTGGCA | egl-43 sgRNA ZD2 |
| *zh119[gfp(frt)::mcm-7]* | CCACCACTTACAACACAGAC | mcm-7 sgRNA2 |
