# Supplementary material for: The Caenorhabditis elegans homolog of the Evi1 proto-oncogene, egl-43, coordinates G1 cell cycle arrest with pro-invasive gene expression during anchor cell invasion
Source: PLoS Genet. 2020 Mar 23;16(3):e1008470. doi: 10.1371/journal.pgen.1008470 (PMC7117773; doi:10.1371/journal.pgen.1008470)
Supplement: S1 Text — (DOCX) [file pgen.1008470.s011.docx]

# Extended methods

# *Generation of reporters and rescue transgenes*

For all constructs, PCR fragments were amplified with Phusion DNA Polymerase (New England Biolabs) and were assembled by Gibson cloning (assembly kit #E2611, New England Biolabs). Details on the constructions of the plasmid and sequences of the oligonucleotides used can be found in **S2 and S3 Tables**. All plasmids were verified by DNA sequencing. For each transgenic reporter, single-copy transgene insertions were created according to the MosSCI protocol by microinjection of 50 ng/µl of the respective reporter plasmid together with 50 ng/µl pJL43.1 (*mos-1* transposase) and 2.5 ng/µl pCFJ90 (*myo-2>mCherry*), 5 µ pcFJ104 (*myo-3>mCherry*) and 10 ng/µl pGH8 (*rap-3>mCherry*) as co-injection markers as described [41].

# *Generation of endogenous reporters/deletions by CRISPR/Cas9*

# The CRISPR/Cas9 method described in [16] was used to generate the endogenous *egl-43* reporters and domain/motif deletions. For the ∆FRE reporter, a 11 bp (TTACTCATCTT) deletion was introduced into the repair template plasmid (pTD34). For the ΔPR and ΔZF1 domain deletions, two sets of guide RNAs each targeting one deletion breakpoint were used. A list of the sgRNAs used for each CRISPR/Cas9 allele can be found in S4 Table.

*EGL-43 ChIPseq analysis*

To produce synchronized L3 larvae of the *egl-43::gfp* strain, gravid adults were bleached, and embryos were left to hatch in M9 buffer overnight at 20°C. The following day, 15’000 L1 larvae per 10cm NGM plate (around 100 plates per experiment) were incubated at 25°C for 27 hours until they had reached the L3 stage. The correct stage was verified by examining under Nomarski optics if the VPCs had reached the Pn.pxx stage in most animals. The L3 larvae were collected by sucrose-flotation, washed with PBS containing protease inhibitors and frozen by dripping the concentrated solution of larvae into liquid nitrogen. Two independently generated populations of L3 larvae were separately processed for ChIP-seq.

# Chromatin immunoprecipitations and library preparations were conducted as described in [42], using formaldehyde and EGS as fixatives and anti-GFP antibody ab290 (Abcam). ChIP-seq reads were aligned to the ce11 assembly of the *C. elegans* genome using BWA v. 0.7.7 [43] with default settings (BWA-backtrack algorithm). The SAMtools v. 0.1.19 ‘view’ utility was used to convert the alignments to BAM format. Normalized ChIP-seq coverage tracks were generated using the BEADS algorithm [44]. MACS2 [45] was used to call peaks with 0.05 FDR cutoff separately on each replicate. Regions with overlapping peaks found in both replicates were kept; as some regions contained more than one clear peak, they were split into separate peaks as in Janes et al, 2018 [46] and https://github.com/jurgjn/yapc by identifying concave regions (those with negative smoothed second derivative) in the averaged BEADS normalized BigWig track, keeping those with curvature scores over 50 and thresholding at the genome-wide level of background in peak neighbourhoods (BEADS score of 2.76). Peaks overlapping blacklisted regions (https://gist.githubusercontent.com/Przemol/ef62ac7ed41d3a84ad6c478132417770/raw/56e98b99e6188c8fb3dfb806ff6f382fe91c27fb/CombinedBlacklists.bed) were discarded.

# Peaks were assigned to genes if they overlapped a promoter or enhancer assigned to a gene in [46]. Peaks that did not overlap a mapped promoter or enhancer were assigned to genes if they overlapped with a gene body or were within 500 bp upstream of a Wormbase TSS sourced from Ensembl v90. Peaks that did not fulfil either criterion were left unassigned. Peak calls and gene assignments are shown in S5 Table. The ChIP-seq data generated in this study are available at the NCBI Gene Expression Omnibus (GEO) (http://www.ncbi.nlm.nih.gov/geo/) under accession number GSE144292.

**Additional references cited in the extended methods**

41. Frøkjær-Jensen C, Davis MW, Hopkins CE, Newman BJ, Thummel JM, Olesen S-P, et al. Single-copy insertion of transgenes in Caenorhabditis elegans. 2008;40: 1375–1383. doi:10.1038/ng.248

42. McMurchy AN, Stempor P, Gaarenstroom T, Wysolmerski B, Dong Y, Aussianikava D, et al. A team of heterochromatin factors collaborates with small RNA pathways to combat repetitive elements and germline stress. Elife. eLife Sciences Publications Limited; 2017;6: 7931. doi:10.7554/eLife.21666

43. Li H, Durbin R. Fast and accurate short read alignment with Burrows-Wheeler transform. Bioinformatics. 2009;25: 1754–1760. doi:10.1093/bioinformatics/btp324

44. Cheung M-S, Down TA, Latorre I, Ahringer J. Systematic bias in high-throughput sequencing data and its correction by BEADS. Nucleic Acids Res. 2011;39: e103–e103. doi:10.1093/nar/gkr425

45. Zhang Y, Liu T, Meyer CA, Eeckhoute J, Johnson DS, Bernstein BE, et al. Model-based analysis of ChIP-Seq (MACS). Genome Biol. BioMed Central; 2008;9: R137–9. doi:10.1186/gb-2008-9-9-r137

46. Jänes J, Dong Y, Schoof M, Serizay J, Appert A, Cerrato C, et al. Chromatin accessibility dynamics across C. elegans development and ageing. Elife. 2018;7: 255. doi:10.7554/eLife.37344
