# Supplementary material for: The Caenorhabditis elegans homolog of the Evi1 proto-oncogene, egl-43, coordinates G1 cell cycle arrest with pro-invasive gene expression during anchor cell invasion
Source: PLoS Genet. 2020 Mar 23;16(3):e1008470. doi: 10.1371/journal.pgen.1008470 (PMC7117773; doi:10.1371/journal.pgen.1008470)
Supplement: Attachment — Submitted filename: Deng_etal_review.pdf [file pgen.1008470.s012.pdf]

Summary:

The research described in this manuscript examined the function of *egl-43* in regulating *C. elegans* anchor cell (AC) invasion. Using CRISPR/Cas9 mediated genome editing, the authors have engineered several new *egl-43* alleles that provide valuable insight into *egl-43* function. Specifically, they identify the long isoform (*egl-43L*) as the predominant isoform functioning during AC invasion as well as regulation of *egl-43* by *fos-1* via a *cis*-regulatory FOS-Responsive Element. Using multiple cell cycle reporters, the authors also show that *egl-43* is required for the AC to undergo G1 cell cycle arrest, and that *egl-43* depletion results in proliferative ACs. The authors claim that this cell cycle dependent function of *egl-43* is independent of the *nhr-67* / CKI-1 pathway known to be involved in AC invasion, although I feel that this is not well-supported by the evidence currently provided in the manuscript. Opportunities to include more convincing controls and to quantify data were sometimes missed by the authors, though should be easily remedied by either better quantification of existing data or new experiments. Suggested revisions to the manuscript include validation of RNAi reagents, explicit indication of sample sizes, and additional experiments (outlined below). Despite the fact that some of this data (i.e., the relationship between *egl-43* and *fos-1*; *egl-43*'s role in cell cycle arrest) has been recently shown in a bioRxviv preprint (Medwig-Kinney et al. (2019)), I feel that the mechanistic insights gained through the authors' careful dissection of the *egl-43* locus is complementary and I am enthusiastic about seeing this work published. Furthermore, the novel finding that ectopic expression of Notch intracellular domain is sufficient to induce proliferation in a normally post-mitotic differentiated cell is a very exciting finding and would be of interest to a broad readership.

Comments:

General comments:

Assuming PLoS Genetics allows for citations of preprints, given the nature of overlap between this work and that of a recently updated bioRxviv preprint (Medwig-Kinney et al. (2019)), I think it would be useful for the field if the authors discussed their results in the context of data showing that *egl-43* regulates both *hlh-2* and *nhr-67* in a cell-cycle dependent manner as well as feedback between EGL-43 and FOS-1. The main discrepancy between this work and Medwig-Kinney et al. (2019) is whether or not *egl-43* and *nhr-67* function independently of each other in mediating G1/G0 arrest in the AC. See below for specific experimental suggestions that might clear this discrepancy up. I would recommend for showing single channel fluorescence images to use grey scale, which the human eye can see subtle differences in easier than false colored images, and only use false colored images for overlays.

In the results and brief Discussion section the authors miss the chance to put their data using endogenously-tagged alleles in the context of what has been shown by their and other labs previously using transgenes - for example, autoregulation of *egl-43* has been shown multiple times based on transgenes and the first potential explanation for this is that levels of *egl-43* are extremely important - see Wang et al. 2014 (doi: 10.1016/j.bbrc.2014.08.049) - where they show that *egl-43* functions through an incoherent feed forward circuit with negative feedback in regulating MIG-10 levels in the AC.

For the most part, the authors represent fluorescence quantification data through box plots, which depict median values. However, given the wide spread of some of this data (e.g., Fig. 4B), median may not be the best statistic to show. I would recommend using an alternative method of data visualization, such as violin plots including mean values and standard deviation.

Introduction:

Potential typos (minor):

"selected" → "select" (paragraph 1)

"trackable" → "tractable" (paragraph 1)

"EGl-43" → "EGL-43" (paragraph 3)

"the VU cell undergoes three rounds of cell divisions" - This is not 100% accurate, as the ρ cells undergo an extra round of division. See Newman, White, & Sternberg (1996).

Results:

"FRT" should be defined upon 1st use of the acronym. The FRT experiments are really elegant - I'm wondering, is the reduced penetrance in these lines as compared to RNAi due to produrance of the protein during the length of time it takes for the flipase to remove the genomic region flanked by FRT sites? I couldn't tell from the images - it looks like there is no expression, but it would be useful to quantify this. "We found no obvious difference in the expression pattern of the two *egl-43* reporters, suggesting that the long *egl-43L* isoform accounts for most of the expression observed." - This claim can be supported by evidence showing quantitative comparison of expression levels in both reporters (not directly provided).

How was the *egl-43Si* RNAi construct validated? The targeting sequence is presumably much smaller (although this information is missing from the supplement) than typical RNAi constructs, so the efficiency may be significantly lower. Also, how did the authors determine the 5' UTR sequence, as I could not find it annotated on WormBase? The authors also may want to consider that there is evidence (Bosher et al., 1999) that RNAi can act on pre-mRNA, which would indicate that this construct may recognize the introns of pre-spliced *egl-43L* transcripts.

Figure 1B-C: It would be helpful to see quantification of this data presented as well.

How was the sample size for the *egl-43L* RNAi vs. *egl-43* RNAi experiment (Results paragraph #2) determined? Typically a minimum sample size of 28 is required to perform a significance test at $\alpha = 0.05$.

Figure 2: The number of animals observed with the representative phenotype shown, with respect to the total number of animals observed, should be indicated in Figures 2A,C-D. The n indicated in the bar graph in panel B is difficult to read due to the small font size (and I expect the font size would need to be increased for publication per journal standards anyway).

The source of the RNR-1::GFP strain/construct (Park & Krause, 1999) should be cited in addition to the WormBook chapter.

Is the characterization of the endogenously-tagged MCM-7::GFP described elsewhere? I know that the transgene has been used as a reporter for actively cycling cells by the van den Heuvel lab (I would recommend citing the data paper, Korzelius et al. 2011, rather than the wormbook chapter here). If this is the first description of the endogenous MCM complex as a reporter for S-phase onset/cycling cells it would be worth characterizing it first and then using it as a reporter. I believe the data, I just think it would be nice to highlight that it's a GFP-knock in - you could cross the allele into the MCM-4::mCherry transgene from the van den Heuvel lab and

just demonstrate that they show the same exact pattern of localization in a cell cycle-dependent way as a supplemental figure?

The original CDK biosensor citation should be included as well from Spencer et al. 2011 when citing its use as it was co-opted from mammalian cell culture.
Image quantification: The Materials and Methods section is specifically lacking a description of how the CDK sensor was quantified, and in general more information is needed in reference to image quantification for all of the data in the manuscript - "built-in measurement tools" in Fiji/ImageJ could mean many different things - how did the authors correct for background/camera noise? Were measurements made from single confocal z-planes? Are the authors' reported mean grey values or integrated density (either is fine, just more details are needed). Did the authors use thresholding and the wand tool to select the region of interest, or did they hand draw regions of interest?

In the updated Medwig-Kinney et al. pre-print, it is shown that the regulatory relationships between *egl-43* and *nhr-67* do not exist until post-AC-specification. I recognize that this data was not available at the time of submission. However, this could explain why the authors do not see a significant change in *nhr-67::GFP* expression in the AC following *egl-43(RNAi)*. More importantly, however, I would suggest that the authors examine mitotic ACs for regulation of gene expression rather than looking at earlier stages, as it is impossible to know whether or not an AC is out of cycle (beyond using a second set of reporters for cell cycle state) so you can not assess whether the single AC you are measuring is going to invade or not. As to the authors' statement that the proliferation of the AC results in dilution of protein expression - this data exists. I would point the authors to data using the transgene containing the full (~5kb) *cdh-3* promoter fused to GFP. In Matus et al. (2015), I found that this promoter was expressed at ~97% of wild-type levels in proliferating ACs (see Figure 4B from Matus et al. 2015), while other reporters are clearly down-regulated, suggesting that GFP is not simply diluted as ACs become mitotic and proliferate but that the actual transcriptional program is changing due to inappropriate cell cycle entry.

This also brings up an important point on the use of the *cdh*-3 promoter for driving constructs of interest in the AC. We have found that the smaller ACEL used to drive *cdh-3* (~1.5kb) for many of the transgenes from the Sherwood lab, also used in this paper (qyIs23 and qyIs50), is regulated by *nhr-67* and *egl-43*, which is why we used the full *cdh-3* promoter (~5kb) to generate new AC reporters for studying *nhr-67* loss of function in Matus et al. 2015. I bring this up because if anything, the use of the smaller *cdh-3*>constructs could under-report the number of ACs due to depletion of the promoter. It took a little digging for me to figure out that the new constructs were designed with the full *cdh-3* promoter, so it would be helpful to distinguish this in the text/methods. We used *cdh-3*$^{1.5}$ vs *cdh-3* in our original paper if that nomenclature is helpful.

Figure 3G,I: The overexpression of CKI-1 in a lineage should cause G1/G0 arrest. It would appear that the authors are making the claim that *egl-43* mediates cell cycle arrest independent of CKI-1, but a more parsimonious explanation would be that depletion of *egl-43* results in downregulation of the *cdh-3* promoter driving CKI-1 expression, and in cases where you see multiple ACs, those ACs do not have a critical threshold of CKI-1 activity to prevent cell cycle entry. One suggestion would

be to quantify levels of CKI-1 in all of the animals and see if there is a statistical correlation between CKI-1 levels and number of ACs observed. While, the 2 AC phenotype could be the result of perturbing AC/VU specification, 3+ ACs shouldn't be observed if the *cdh-3*>CKI-1 is functioning 100% of the time.

Figure 3G-I: The key says "control siblings" - does this mean that these are the progeny resulting from a cross? I assume not, but this terminology may be misleading and whether animals of homozygous or heterozygous is an important distinction.

Figure 4A: This data should be quantified. Also how was expression of *lin-12* determined? How were the boundaries of ACs versus VUs determined in adjacent cells? The endogenously- tagged *lin-12::GFP* reporter from Attner et al. (2019) has both membrane bound and nuclear localization, making it easier to distinguish which cell has active Notch signaling - this strain might be easier to use and would better make this really stunning point that active Notch signaling post-AC/VU decision can force the differentiated AC into the cell cycle and inhibit invasion.

Figures 4A-C: When was *lin-12* RNAi treatment administered? Knockdown of *lin-12* prior to AC/VU specification may confound the number of ACs observed. It would be worthwhile to try an L2 plating of *lin-12(RNAi)* and see if, at some penetrance, you can repeat your experimental results.

Figure 4D: What percentage of animals are the phenotypes shown indicative of?

Figure 5B: It would be helpful to show GFP::EGL-43L expression without *fos-1(ar105)* in the background here.

The authors postulate that *egl-43* has cell cycle dependent (*lin-12*) and independent (*fos-1*) roles, but this is not supported by the data showing that *lin-12(RNAi)* rescues AC invasion in *egl-43(RNAi)* animals.

The authors should mention the potential effects that the endogenous transcriptional reporters (with pre-floxed SEC) have on protein function.

The introduction and discussion need elaboration, specifically with regard to links between Notch signaling and cell cycle regulation, in *C. elegans* and other model systems.

The authors argue that *egl-43* and *nhr-67* control cell cycle arrest in distinct pathways. To show this convincingly, they should perform the *lin-12*/Notch experiments with *nhr-67* RNAi perturbation experiments, the expectation would be that *nhr-67(RNAi)* does not induce *lin-12* expression if the two pathways are independent. Alternatively, as we believe that *egl-43* does regulate *nhr-67* activity, it would be interesting if this was still the case - that *nhr-67(RNAi)* does not regulate *lin-12*, as we have recently shown that endogenous *lin-12::GFP* is strongly down-regulated pre-AC/VU decision in our bioRxiv preprint. If you find that *nhr-67(RNAi)* doesn't turn on *lin-12::GFP*, it could also suggest that *egl-43* has *nhr-67*-dependent and *nhr-67*-independent roles in maintaining the AC in a post-mitotic state, and provide an explanation why *nhr-67(RNAi)* on an *nhr-67(pf88)* hypomorphic allele

doesn’t significantly increase the AC invasion/proliferation defect (it makes it slightly worse, but there are still a small population of ACs that invade).

Supplementary tables:

Tables S2 and S3 is missing the plasmids and primers used to generate the *egl-43Si* and *egl-43Li* RNAi constructs. Information regarding the targeting sequences used would also be helpful to include.

Table S2 contains primers whose sequences are not provided in Table S3. Namely oTD140-143.

Table S3 contains sequences of primers that are not defined in Table S2 or elsewhere. Namely the OEL316-319.
