# Supplementary material for: The Caenorhabditis elegans homolog of the Evi1 proto-oncogene, egl-43, coordinates G1 cell cycle arrest with pro-invasive gene expression during anchor cell invasion
Source: PLoS Genet. 2020 Mar 23;16(3):e1008470. doi: 10.1371/journal.pgen.1008470 (PMC7117773; doi:10.1371/journal.pgen.1008470)
Supplement: Attachment — Submitted filename: Point-by-point response.pdf [file pgen.1008470.s013.pdf]

Point-by-point response

(original comments by the reviewers are in italic)

To Reviewer #1:

I have no major criticisms of this work. It appears solid and complete, and the text is well-written and clear. The only thing I felt was lacking was more discussion of the role of egl-43 in the fate decision of the AC as compared to other studies on this subject, in particular the recent work from the Greenwald lab (HLH-2/E2A Expression Links Stochastic and Deterministic Elements of a Cell Fate Decision during C. elegans Gonadogenesis, Current Biology, Sept 2019). How does egl-43 function fit in with the HLH-2 expression clock described in this paper?

We have added the new reference on HLH-2 to the introduction section. We did not discuss this aspect because this manuscript focusses on the role of EGL-43 during AC invasion, while HLH-2 is involved in the AC/VU decision.

Response to reviewer #2:

Assuming PLOS Genetics allows for citations of preprints, given the nature of overlap between this work and that of a recently updated bioRxviv preprint (Medwig-Kinney et al. (2019)), I think it would be useful for the field if the authors discussed their results in the context of data showing that egl-43 regulates both hlh-2 and nhr-67 in a cell-cycle dependent manner as well as feedback between EGL-43 and FOS-1.

The main discrepancy between this work and Medwig-Kinney et al. (2019) is whether or not egl-43 and nhr-67 function independently of each other in mediating G1/G0 arrest in the AC. See below for specific experimental suggestions that might clear this discrepancy up.

We are now citing the recent publication by Medwig-Kinnney (ref #34) in the results and discussion sections. Our data are fully consistent with this publication, as discussed on p. 13 and outlined below. In particular, by analyzing later stage larvae we could also observe a mutual regulation of *egl-43* and *nhr-67* (Fig 3). Though, our new data on the *lin-12* pathway further distinguish between the roles of EGL-43 and NHR-67 in the control of AC proliferation as explained below (p.13 & Fig. 7).

In the results and brief Discussion section the authors miss the chance to put their data using endogenously-tagged alleles in the context of what has been shown by their and other labs previously using transgenes - for example, autoregulation of egl-43 has been shown multiple times based on transgenes and the first potential explanation for this is that levels of egl-43 are extremely important - see Wang et al. 2014 (doi: 10.1016/j.bbrc.2014.08.049) - where they show that egl-43 functions through an incoherent feed forward circuit with negative feedback in regulating MIG-10 levels in the AC.

The reference to Wang et al. 2014 (ref #33) is now included, in particular because our new ChIP-seq analysis supports direct binding of EG-43 to *mig-10* (suppl. Fig. S6B). In the discussion section, we wanted to focus on the *egl-43-fos-1* feedback loop since this aspect is relevant for our work (p.13). The importance of *egl-43* levels in this context is discussed (p.14, bottom).

For the most part, the authors represent fluorescence quantification data through box plots, which depict median values. However, given the wide spread of some of this data (e.g., Fig. 4B), median may not be the best statistic to show. I would recommend using an alternative method of data visualization, such as violin plots including mean values and standard deviation.

All box plots in all figures have now been replaced with scatter plots showing each data point, the mean values and standard deviations. Statistical tests for quantitative measurements are included.

Potential typos (minor):

"selected" → "select" (paragraph 1)

"trackable" → "tractable" (paragraph 1)

"EGl-43" → "EGL-43" (paragraph 3)

All typos have been corrected.

"the VU cell undergoes three rounds of cell divisions" - This is not 100% accurate, as the ρ cells undergo an extra round of division. See Newman, White, & Sternberg (1996).

This sentence has been corrected (p.4).

Results:

"FRT" should be defined upon 1st use of the acronym. The FRT experiments are really elegant - I'm wondering, is the reduced penetrance in these lines as compared to RNAi due to perdurance of the protein during the length of time it takes for the flipase to remove the genomic region flanked by FRT sites? I couldn't tell from the images - it looks like there is no expression, but it would be useful to quantify this.

"FRT" has been defined at its first use (p.5). Perdurance of the protein as one possible explanation is mentioned as a possible explanation, especially since low EGL-43 expression in the AC was detectable in some of the animals after FLP-out (end of p.6).

"We found no obvious difference in the expression pattern of the two egl-43 reporters, suggesting that the long egl-43L isoform accounts for most of the expression observed." - This claim can be supported by evidence showing quantitative comparison of expression levels in both reporters (not directly provided).

Suppl. Fig. S2 shows a quantification of the different reporter expression levels including the levels in the RNAi experiments. Note that we have quantified the N- and C-terminally tagged EGL-43 reporters separately because the site of GFP insertion could affect the signal levels (legend to Fig. S2).

How was the egl-43Si RNAi construct validated? The targeting sequence is presumably much smaller (although this information is missing from the supplement) than typical RNAi constructs, so the efficiency may be significantly lower. Also, how did the authors determine the 5' UTR sequence, as I could not find it annotated on WormBase?

We have designed an RNAi clone targeting the 96 bp 5'UTR of *egl-43S* located in the intron of *egl-43L*. The 5'UTR sequence can be found in the RNAseq data accessible through wormbase. The details on the construction and sequence of the RNAi vector are now included in suppl. Tab S2&S3.

The reviewer is right in his comment that a negative RNAi experiment by itself is inconclusive. Though, *egl-43L* RNAi caused a complete loss of AC expression, which is a good argument against any <u>detectable</u> AC expression of EGL-43S. To further address this point in the best possible way, we used genome editing. It should be noted that not only the *egl-43LΔS::gfp allele,* which specifically deletes the initiation codon of the S isoform, showed no invasion defect, but also the *egl-43LΔZF::gfp* allele, which deletes the *egl-43S* promoter region (p7, 2nd para). Together, we think our data make a strong case against any role of the S isoform during AC invasion.

Figure 1B-C: It would be helpful to see quantification of this data presented as well.

Numbers have been added to the figure and figure legend and quantification of expression levels is shown in suppl Fig S2. The quantification of the exact AC numbers is shown in figure 2B using the *cdh-3>mCherry::PH* reporter outlining the ACs and GFP::MCM-7 labeling the AC nuclei.

How was the sample size for the egl-43L RNAi vs. egl-43 RNAi experiment (Results paragraph #2) determined? Typically a minimum sample size of 28 is required to perform a significance test at a = 0.05.

We are now showing the combined numbers obtained in two independent RNAi experiments performed with the same genetic background. This is explicitly mentioned on p.6, 2nd para. The two experiment individually showed a similar penetrance of the phenotypes.

Figure 2: The number of animals observed with the representative phenotype shown, with respect to the total number of animals observed, should be indicated in Figures 2A,C-D. The n indicated in the bar graph in panel B is difficult to read due to the small font size (and I expect the font size would need to be increased for publication per journal standards anyway).

The numbers indicating the observed frequencies have been added to the figure legends and changes haven been made as suggested.

The source of the RNR-1::GFP strain/construct (Park & Krause, 1999) should be cited in addition to the WormBook chapter.

This reference has been included (#23).

Is the characterization of the endogenously-tagged MCM-7::GFP described elsewhere? I know that the transgene has been used as a reporter for actively cycling cells by the van den Heuvel lab (I would recommend citing the data paper, Korzelius et al. 2011, rather than the wormbook chapter here). If this is the first description of the endogenous MCM complex as a reporter for S-phase onset/cycling cells it would be worth characterizing it first and then using it as a reporter. I believe the data, I just think it would be nice to highlight that it's a GFP-knock in - you could cross the allele into the MCM-4::mCherry transgene from the van den Heuvel lab and just demonstrate that they show the same exact pattern of localization in a cell cycle-dependent way as a supplemental figure?

We have added the information on the generation of the mcm-7 reporter (suppl. Tab S2&S3) and the Korzelius et al reference (p.7, #21).

The original CDK biosensor citation should be included as well from Spencer et al. 2011 when citing its use as it was co-opted from mammalian cell culture. Image quantification: The Materials and Methods section is specifically lacking a description of how the CDK sensor was quantified, and in general more information is needed in reference to image quantification for all of the data in the manuscript - "built-in measurement tools" in Fiji/ImageJ could mean many different things - how did the authors correct for background/camera noise? Were measurements made from single confocal z-planes? Are the authors' reported mean grey values or integrated density (either is fine, just more details are

needed). Did the authors use thresholding and the wand tool to select the region of interest, or did they hand draw regions of interest?

The Spencer et al reference has been added (p.7) and the quantification is now described in the methods section (p.17).

In the updated Medwig-Kinney et al. pre-print, it is shown that the regulatory relationships between egl-43 and nhr-67 do not exist until post-AC-specification. I recognize that this data was not available at the time of submission. However, this could explain why the authors do not see a significant change in nhr-67::GFP expression in the AC following egl-43(RNAi). More importantly, however, I would suggest that the authors examine mitotic ACs for regulation of gene expression rather than looking at earlier stages, as it is impossible to know whether or not an AC is out of cycle (beyond using a second set of reporters for cell cycle state) so you can not assess whether the single AC you are measuring is going to invade or not…l.

This is the only point where there was a discrepancy between our data and the Medwig-Kinney et al publication. To further investigate this issue, we compared -as suggested- expression levels in early (Pn.p stage) and mid-L3 (Pn.pxx stage) larvae. Indeed, at the later stage, we could also observe reduced expression, suggesting a mutual regulation of *egl-43* and *nhr-67*. In the revised manuscript, we now show the reduction at the later stage in Fig. 3A-D and the earlier (Pn.p) stage in suppl. Fig s3.

Figure 3G,I: The overexpression of CKI-1 in a lineage should cause G1/G0 arrest. It would appear that the authors are making the claim that egl-43 mediates cell cycle arrest independent of CKI-1, but a more parsimonious explanation would be that depletion of egl-43 results in downregulation of the cdh-3 promoter driving CKI-1 expression, and in cases where you see multiple ACs, <u>those ACs do not have a critical threshold of CKI-1 activity to prevent cell cycle entry.</u> One suggestion would be to quantify levels of CKI-1 in all of the animals and see if there is a statistical correlation between CKI-1 levels and number of ACs observed. While, the 2 AC phenotype could be the result of perturbing AC/VU specification, 3+ ACs shouldn't be observed if the cdh-3>CKI-1 is functioning 100% of the time.

Only worms showing clear mNG expression in the AC from the cdh-3>CKI-1::SL2::mNG transgene were scored (legend to Fig. 3), and we did not observe a reduction in mNG expression when comparing *egl-43* to *nhr-67* RNAi (Fig. 3E). Thus, the different sensitivity to CKI-1 overexpression cannot be explained by an effect on the *cdh-3* promoter. As stated in the manuscript, there is a slight effect of CKI-1 on the AC number in *egl-43* RNAi animals (Fig. 3G, 81% vs 72% more than 2 AC, thus excluding AC-VU specification defects, as pointed out). Together with the new data showing that *lin-12* RNAi only weakly suppresses the *nhr-67* phenotypes (Fig. 4 and see below), the CKI-1 overexpression experiment supports our conclusion that NHR-67 inhibits AC proliferation <u>mainly</u> via CKI-1, while EGL-43 acts <u>predominantly</u> by repressing *lin-12* expression. It is not an all or none effect, as CKI-1 has a weak effect on *egl-43* RNAi, and *lin-12* RNAi does partially suppress AC proliferation in NHR-67 RNAi (Fig. 4C). Regarding the underlying mechanism, we can only speculate that the relative levels between CKI-1 and CDK activity must differ between *nhr-67* and *egl-43* RNAi. One could for example imagine that increased *lin-12* signaling caused by *egl-43* RNAi causes a hyperactivation of CDK/Cyclin, which overcomes CKI-1 inhibition, while NHR-67 acts directly predominantly by regulating CKI-1 expression. We think, a threshold model may be an appropriate way of looking at this effect. *nhr-67* inactivation reduces the threshold set by

decreasing CKI-1 levels, whereas *egl-43* inactivation overcomes the threshold by increasing LIN-12 signaling (see discussion 1&2nd[st] para p.14).

Figure 3G-I: The key says "control siblings" - does this mean that these are the progeny resulting from a cross? I assume not, but this terminology may be misleading and whether animals of homozygous or heterozygous is an important distinction.

Control siblings refers to the siblings that lacked the extrachromosomal CKI-1 transgene. This is now explained in the legend to fig. 3.

Figure 4A: This data should be quantified. Also how was expression of lin-12 determined? How were the boundaries of ACs versus VUs determined in adjacent cells? The endogenously-tagged lin-12::GFP reporter from Attner et al. (2019) has both membrane bound and nuclear localization, making it easier to distinguish which cell has active Notch signaling - this strain might be easier to use and would better make this really stunning point that active Notch signaling post-AC/VU decision can force the differentiated AC into the cell cycle and inhibit invasion.

Numbers have been added in the Figure 4A legend. Expression of *lin-12* was determined by analyzing z-stacks across the AC. In combination with the AC membrane marker *cdh-3>mCherry::PH*, the fosmid-based *lin-12* reporter used in our study can easily distinguish between AC and VU expression.

Figures 4A-C: When was lin-12 RNAi treatment administered? Knockdown of lin-12 prior to AC/VU specification may confound the number of ACs observed. It would be worthwhile to try an L2 plating of lin-12(RNAi) and see if, at some penetrance, you can repeat your experimental results.

lin-12 RNAi was applied at the same time as the other RNAi at the L1 stage. Since the penetrance of AV/VU defects caused by *lin-12* RNAi alone is low (2/122), the timing of RNAi likely does not matter much. In addition, we show the fraction of animals with more than 2ACs

Figure 4D: What percentage of animals are the phenotypes shown indicative of?

Numbers have been added to the figure legend.

Figure 5B: It would be helpful to show GFP::EGL-43L expression without fos-1(ar105) in the background here.

EGL-43 expression in the *fos-1(ar105)* heterozygote siblings is the better control since it excludes possible effects caused by the genetic background. Expression in a wild-type background is shown in Fig. 1.

The authors postulate that egl-43 has cell cycle dependent (lin-12) and independent (fos-1) roles, but this is not supported by the data showing that lin-12(RNAi) rescues AC invasion in egl-43(RNAi) animals.

lin-12 RNAi did not fully rescue the AC invasion defects of *egl-43* RNAi animals. About 14% (i.e. 19/135) animals treated with *egl-43; lin-12* double RNAi had an invasion defect, and eight

of these 19 animals had a single AC (p 10, 1st para). Thus, AC proliferation and invasion do not always correlate in *egl-43* RNAi animals.

The new ChIP-seq data (Fig. 6) further support a function of EGL-43 in regulating *fos-1*, and the data in suppl Fig S5 show that *fos-1* does not regulate the cell cycle and that *fos-1* is not regulated by *lin-12*. Moreover, the ChIP-seq analysis indicated that the regulation of *mig-10* expression by EGL-43 might by direct. Together, we think this is good evidence for a cell-cycle independent function of EGL-43.

The authors should mention the potential effects that the endogenous transcriptional reporters (with pre-floxed SEC) have on protein function.

This is now explained (p12).

The introduction and discussion need elaboration, specifically with regard to links between Notch signaling and cell cycle regulation, in C. elegans and other model systems.

The discussion has been modified to include these aspects, see end of p. 13 to p.14

The authors argue that egl-43 and nhr-67 control cell cycle arrest in distinct pathways. To show this convincingly, they should perform the lin-12/Notch experiments with nhr-67 RNAi perturbation experiments, the expectation would be that nhr-67(RNAi) does not induce lin-12 expression if the two pathways are independent. Alternatively, as we believe that egl-43 does regulate nhr-67 activity, it would be interesting if this was still the case - that nhr-67(RNAi) does not regulate lin-12, as we have recently shown that endogenous lin-12::GFP is strongly down-regulated pre-AC/VU decision in our bioRxiv preprint. If you find that nhr-67(RNAi) doesn't turn on lin-12::GFP, it could also suggest that egl-43 has nhr-67-dependent and nhr-67-independent roles in maintaining the AC in a post-mitotic state, and provide an explanation why nhr-67(RNAi) on an nhr-67(pf88) hypomorphic allele doesn't significantly increase the AC invasion/proliferation defect (it makes it slightly worse, but there are still a small population of ACs that invade).

We have addressed this point by examining LIN-12::GFP expression after NHR-67 RNAi, at the same stage (Pn.pxx) as for *egl-43* RNAi and by testing for a suppression of the *nhr-67* proliferation and invasion defects by *lin-12* RNAi. While *nhr-67* RNAi did increase *lin-12* expression in the AC (Fig. 4A), *lin-12* RNAi only slightly suppressed the proliferation defect and did not significantly rescue the *nhr-67* AC invasion defect (Fig. 4B,C). This experiment essentially mirrors the differential effects of CKI-1 overexpression on *egl-43* and *nhr-67* (Fig. 3, as discussed on p. 14, 2nd para). See also our response to the comments on the CKI-1 experiment (Fig. 3 G,I) above.

Supplementary tables:

Tables S2 and S3 is missing the plasmids and primers used to generate the egl-43Si and egl-43Li RNAi constructs. Information regarding the targeting sequences used would also be helpful to include.

Table S2 contains primers whose sequences are not provided in Table S3. Namely oTD140-143.

Table S3 contains sequences of primers that are not defined in Table S2 or elsewhere. Namely the OEL316-319.

All the changes have been made as suggested. Primers that were not used have been removed and new primers for the additional constructs were added.

Response to Reviewer #3:

Specific points:

There are some "rigor and reproducibility" issues in the data presentation that need to be addressed:

We have added the numbers of phenotype per animals observed to all figure legends and quantification of fluorescence intensities where possible. Statistical analysis were done for all quantitative measurements.

Fig 1B quantification: how many animals were examined, how many showed these expression patterns, and how many had multiple ACs and/or no invasion?

Numbers of worms examined in Fig 1B&C have been added to the figure legend. All animals examined showed essentially the same expression pattern (i.e. AC expression), which is typical of endogenous reporters. None had multiple ACs or invasion defects, which is now described in the manuscript and figure legend.

Quantification of the AC expression levels is shown in suppl. Fig S2A&B. We quantified the levels of N- and C-terminal GFP fusions separately since the site of GFP insertion can affect signal strength (see legend to suppl. Fig S2).

Fig 2B: statistics? Are the changes over time significant?

Statistic tests are now included in figure 2.

Fig 2C and 2D quantification: how many animals were examined, how many showed these expression patterns?

All the numbers have been added in to the figure legend.

Figure 3H and 3I: What % of animals had multiple ACs? How does this relate to the % that had invasion defects?

Figs 3F and 3G haven been modified as described in our response to reviewer #3. The % of animals with more than two ACs is now indicated in 3 in all graphs showing AC counts -Figs 3G, 4B&F. (>2 excludes potential AC/VU specification defects.) In addition, Figs 3F and 4G show the correlation between invasion defects and AC numbers.

Both, CKI-1 overexpression and *lin-12* RNAi resulted in the development of animals with one AC that failed to invade, indicating that the AC invasion and proliferation phenotypes do not strictly correlate. See also above our response to a similar comment made by reviewer #3 and the modifications on p.9 1st and last para & p.14 2nd para.

Figure 4F and 4G: Are the %s in G based on pooling the 3 lines of each genotype, or are they based on one specific line? What % of the animals scored in G had multiple ACs and is there an absolute correlation between the two types of defects?

Yes, the % invasion defects in Fig 4G are based on pooling the 3 lines (see figure legend). The AC numbers haven been integrated into Fig 4G to show the correlation.

94% of the animals with an invasion defect had more than 1 AC, indicating that the invasion defect of NICD-overexpressing arrays is mainly a consequence of AC proliferation. Interestingly, a small portion (~27%) of worms with multiple ACs managed to invade.

Addressing any of the below would add more meat and impact to this paper, but addressing points 2,3,4 would be especially relevant:

2. Circumstantial evidence suggests that the egl-43 long isoform might be required because it is the predominant one transcribed in the AC, but the authors can't exclude that there is something unique about the protein made by this isoform. Transgenic rescue experiments with each isoform could address this question more definitively.

We have presented more evidences beside the expression of *egl-43*L to argue that it is required. AC specific FLP-out of the *egl-43*L isoform only and specific deletion of the *egl-43*S isoform. To address the requirement of the domains that are specific to the *egl-43*L isoform (the PR the N-terminal Zn fingers), we used genome engineering to introduce in-frame deletions of the PR domain and the *egl-43*L ZF1 domains (Fig. 1A, p7, 1st para). We believe, a domain deletion by genome engineering is at least as conclusive as transgenic rescue experiments. The two *egl-43L*-specific domains (the ZF1 and PR domains) were not required for AC invasion, indicating that the EGL-43L and -S proteins could in principle perform the same function in the AC, but that they rather differ in their AC expression patterns.

3. The authors present more evidence for the previously observed correlation between cell cycle arrest and invasive activity, but the basis for this correlation remains unclear. On p. 10 the authors speculate that "EGL-43 might perform two functions in the AC; first to repress AC proliferation and second to activate pro-invasive gene expression". But data in Figure 4 suggest that EGL-43 might do one thing (inhibit lin-12) to accomplish both results. What is the explanation for why lin-12(gf) suppresses proliferation and invasion defects (Figure 4), but CKI expression mostly does not (Figure 3)? At what step of the cell cycle is lin-12 acting? Does lin-12 affect expression of the mentioned "pro-invasive" genes?

See also above our response to a similar comment made by reviewer #2. In around 14% of *egl-43* and *lin-12* double RNAi animals the AC failed to invade (fig 4), and 8 of these 19 animals had a single AC (p 10, 1st para). Thus, AC proliferation and invasion do not always correlate in *egl-43* RNAi animals. A possible explanation for the different sensitivities of *nhr-67* and *egl-43* to CKI-1 overexpression and *lin-12* RNAi is now discussed on p.13-14.

Moreover the ChIP-seq data (Fig. 6) further support a function of EGL-43 in directly regulating *fos-1* expression, and the data in suppl Fig S5 show that *fos-1* does not regulate the cell cycle and that *fos-1* is not regulated by *lin-12*. Together, we think this is good evidence for a cell-cycle independent function of EGL-43.

What is the explanation for why lin-12(gf) suppresses proliferation and invasion defects (Figure 4), but CKI expression mostly does not (Figure 3)?

We assume the reviewer meant *lin-12(lf)* instead of *lin-12(gf)*. This is now discussed in more detail on p.14 as explained above in our response to a similar comment made by reviewer #2. Since we have found that the *nhr-67* phenotype is much less sensitive to *lin-12* RNAi (see revised Fig. 4), our data suggest that CKI-1 and LIN-12 act through different mechanisms to control the cell cycle of the AC (see updated model in Fig. 7). By regulating CKI-1 expression, NHR-67 sets a threshold for the CDK/Cyclin kinases in the AC (i.e. the ratio of CDK to CKI activity), while the activation of LIN-12 signaling by *egl-43* RNAi overcomes this threshold, possibly by enhancing CDK activity. The involvement of LIN-12 Notch signaling in the cell cycle is discussed on p.13.

Another possibility is that EGL-43 regulates the G1 arrest of the AC not via *cki-1*, but rather via *cki-2*, as Buck et.al (2009) has shown *cki-1* and *cki-2* function in different pathways to achieve cell cycle inhibition. However, we did not include this possibility in the discussion as there exist at present no data to support this claim.

At what step of the cell cycle is lin-12 acting?

This is now briefly discussed on p.13. Notch was shown in different mammalian cell lines to regulate cell cycle progression positively or negatively depending on the cellular context. In many cases, Notch promotes G1-to-S phase transition by either inducing cell cycle activators (e.g, cyclin A, cyclin D, E2f transcriptional factor) or by inducing the degradation of cell cycle inhibitors CIP/KIP. Along these lines, we did observe an activation of cell cycle reporters such as *mcm-7* (Fig. 4) and *cye-1* (not shown) by AC-specific NICD expression.

Does lin-12 affect expression of the mentioned "pro-invasive" genes?

No regulation of *fos-1* expression by LIN-12 NICD was observed (suppl. Fig S5). Since FOS-1 is one essential regulator of the pro-invasive genes (*zmp-1*, cdh-3 etc), we would not expect LIN-12 signaling to directly regulate pro-invasive genes expression. Even if we did observe changes in the expression of the FOS-1 targets caused by NICD expression -which we did not test as we focused on *fos-1*- this would more likely by a consequence of AC over-proliferation rather than a direct effect of *lin-12*.

4. Is the cross-regulation of egl-43 and fos-1 functionally important? Can cdh-3>fos-1 rescue egl-43 defects or vice versa? Or are they both needed in parallel?

We previously tested if *egl-43* overexpression rescued *fos-1* mutants- which it did not (Rimann 2009). R, *egl-43* expression does not strictly depend on FOS-1, pointing at additional input to EGL-43 (see p.14). These findings suggest that, while *fos-1* and *egl-43* positively regulate each other, the two transcription factors likely act in parallel.

5. The paper does not show whether lin-12, fos-1, or any of the other downstream effectors of EGL-43 are direct targets, so it does not provide insights into EGL-43 binding properties or mechanisms of transcriptional regulation (e.g. is EGL-43 both a tx repressor and activator?).

We have now included the results of EGL-43 ChIP-seq analysis, which is consistent with a direct regulation of *egl-43, fos-1* and *lin-12* by EGL-43 (Fig. 6).

6. Given that egl-43 is expressed in both AC and VU, and seems to regulate different target genes in each, what is the basis for target specificity?

The effect of EGL-43 on its targets is probably context-dependent as EGL-43 represses some targets (*lin-12, mig-10*) and activates others (*fos-1* and itself) in the AC. Less is known about EGL-43 targets in the VUs.

Have all data underlying the figures and results presented in the manuscript been provided?
Large-scale datasets should be made available via a public repository as described in the *PLOS Genetics* data availability policy, and numerical data that underlies graphs or summary statistics should be provided in spreadsheet form as supporting information.

Reviewer #1: Yes

Reviewer #2: No: We cannot find information pertaining to the sequence of the egl-43Si 5'UTR used in the generation of the RNAi targeting clone to the short isoform of egl-43. Also, I could not find the data supporting the claim that the different GFP-tagged alleles have the same expression levels in the AC

Primers and strategy used to generate the *egl-43S* RNAi construct are provided in suppl. Tab S2&3. As mentioned above, intensities of N- and C-terminally GFP-tagged *egl-43* alleles are not directly comparable.

Reviewer #3: No: numerical data for bar graphs has not been provided

All numbers have been provided.
