# Supplementary material for: The Caenorhabditis elegans homolog of the Evi1 proto-oncogene, egl-43, coordinates G1 cell cycle arrest with pro-invasive gene expression during anchor cell invasion
Source: PLoS Genet. 2020 Mar 23;16(3):e1008470. doi: 10.1371/journal.pgen.1008470 (PMC7117773; doi:10.1371/journal.pgen.1008470)
Supplement: Attachment — Submitted filename: Response.pdf [file pgen.1008470.s014.pdf]

Response to Dr. Matus:

We have directly addressed your points in the relevant part of the discussion of the data (p.13, last para and p. 14, 1st para) and changed the model figure (Fig. 7) as recommended.

We now explicitly mention the difference between the *cki-1* transgene used in our study and the transgene used by Medwig-Kinney et al. [34], and discuss the possibility that a further increase in CKI-1 levels may also suppress the *egl-43* phenotype. On the other hand, the *cki-1* transgene we used did cause a robust suppression of the *nhr-67* but not the *egl-43* RNAi AC proliferation and invasion phenotypes (Fig. 3F,G). Hence, the model we propose is not black and white, but we rather suggest that *egl-43* is *less sensitive* than *nhr-67* to the increased *cki-1* dosage we used (p.14, end of 1st para).

Regarding a possible direct regulation of *cki-1* by EGL-43, the CHIP-seq peaks in the *cki-1* locus are in the 5' region, which is not sufficient for AC expression (Matus et al, 2015). Moreover, we did not observe a significant effect of *egl-43* RNAi on *cki-1* expression using an endogenous *cki-1::gfp* reporter (Deng et al. unpublished results). Therefore, we speculate that EGL-43 might regulate *cki-1* expression indirectly via NHR-67 (p. 14, end of 2nd para).
